# Particle Swarm Optimisation Applied to the Direct Aperture Optimisation Problem in Radiation Therapy

**DOI:** 10.3390/cancers15194868

**Published:** 2023-10-06

**Authors:** Gonzalo Tello-Valenzuela, Mauricio Moyano, Guillermo Cabrera-Guerrero

**Affiliations:** Escuela de Ingeniería Informática, Pontificia Universidad Católica de Valparaíso, Av. Brasil 2241, Valparaíso 2362807, Chile; gonzalo.tello.v@mail.pucv.cl (G.T.-V.); mauricio.moyano@pucv.cl (M.M.)

**Keywords:** direct aperture optimisation, intensity-modulated radiation therapy, particle swarm optimisation

## Abstract

**Simple Summary:**

Intensity Modulated Radiation Therapy (IMRT) is a cancer treatment that targets cancer cells while protecting nearby healthy organs using a linear accelerator. Traditional IMRT planning involves a sequential process: optimizing beam intensities (Fluence Map Optimization) for a set of angles and then sequencing (Multi-Leaf Sequencing). Unfortunately, treatment plans obtained by the sequencing step are severely impaired. One approach that addresses the problem described is the Direct Aperture Optimisation (DAO) approach. The DAO problem aims at simultaneously determining deliverable aperture shapes and a set of radiation intensities. This approach considers physical and delivery time constraints, allowing clinically acceptable treatment plans to be generated. In this work, we adapt the Particle Swarm Optimisation to solve the DAO and introduce a reparation heuristic to enhance treatment plans. We tested our method on prostate cancer patients and found that it delivers radiation more efficiently than the traditional approach, reducing treatment time and improving outcomes.

**Abstract:**

Intensity modulated radiation therapy (IMRT) is one of the most used techniques for cancer treatment. Using a linear accelerator, it delivers radiation directly at the cancerogenic cells in the tumour, reducing the impact of the radiation on the organs surrounding the tumour. The complexity of the IMRT problem forces researchers to subdivide it into three sub-problems that are addressed sequentially. Using this sequential approach, we first need to find a beam angle configuration that will be the set of irradiation points (beam angles) over which the tumour radiation is delivered. This first problem is called the Beam Angle Optimisation (BAO) problem. Then, we must optimise the radiation intensity delivered from each angle to the tumour. This second problem is called the Fluence Map Optimisation (FMO) problem. Finally, we need to generate a set of apertures for each beam angle, making the intensities computed in the previous step deliverable. This third problem is called the Sequencing problem. Solving these three sub-problems sequentially allows clinicians to obtain a treatment plan that can be delivered from a physical point of view. However, the obtained treatment plans generally have too many apertures, resulting in long delivery times. One strategy to avoid this problem is the Direct Aperture Optimisation (DAO) problem. In the DAO problem, the idea is to merge the FMO and the Sequencing problem. Hence, optimising the radiation’s intensities considers the physical constraints of the delivery process. The DAO problem is usually modelled as a Mixed-Integer optimisation problem and aims to determine the aperture shapes and their corresponding radiation intensities, considering the physical constraints imposed by the Multi-Leaf Collimator device. In solving the DAO problem, generating clinically acceptable treatments without additional sequencing steps to deliver to the patients is possible. In this work, we propose to solve the DAO problem using the well-known Particle Swarm Optimisation (PSO) algorithm. Our approach integrates the use of mathematical programming to optimise the intensities and utilizes PSO to optimise the aperture shapes. Additionally, we introduce a reparation heuristic to enhance aperture shapes with minimal impact on the treatment plan. We apply our proposed algorithm to prostate cancer cases and compare our results with those obtained in the sequential approach. Results show that the PSO obtains competitive results compared to the sequential approach, receiving less radiation time (beam on time) and using the available apertures with major efficiency.

## 1. Introduction

Cancer is a type of disease that causes abnormal growth of cells in the body, leading to the formation of carcinomas, which can eventually turn into malignant tumours. In 2020, the International Agency for Research on Cancer reported 19.3 million new cancer cases and nearly 10 million cancer-related deaths [1]. There are various methods for treating cancer, and the treatment choice largely depends on the specific type of cancer and its impact on the patient’s health.

**Figure 1:**
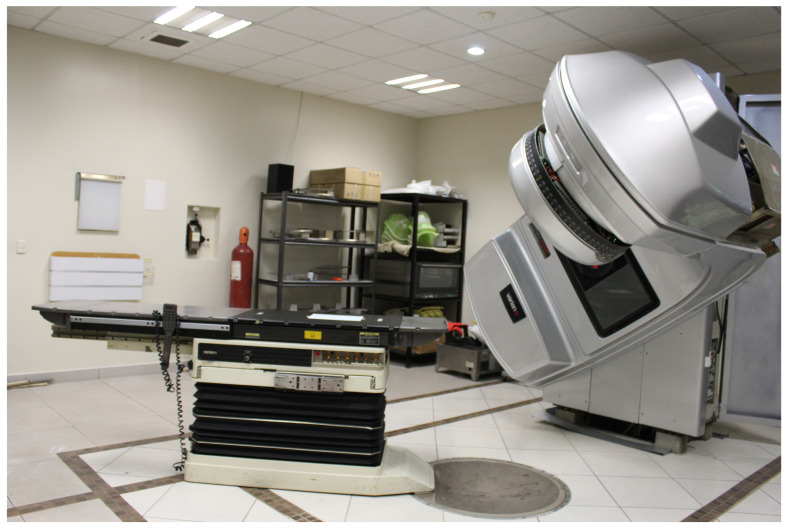
Linear accelerator from the Centro Oncologico Hondureño in Honduras.

Radiotherapy is a commonly used cancer treatment technique involving exposing patients to ionising radiation to target cancerous cells. There are various forms of radiotherapy, such as Volumetric Modulated Arc Therapy (VMAT), Stereotactic Body Radiation Therapy (SBRT), and Intensity Modulated Radiation Therapy (IMRT), among others. IMRT is one of the most widely used methods of radiation therapy, and is delivered using a linear accelerator (linac) machine [2] (Figure 1). IMRT aims to effectively deliver the prescribed radiation dose to the cancerous cells while minimising the exposure of healthy structures [3]. This is achieved by modulating the radiation passing through the linac using a multi-leaf collimator (MLC) device.

The IMRT technique enables the delivery of an optimal radiation dose to the tumour while minimising exposure to surrounding healthy organs [4]. However, finding a treatment plan that balances the desired dose to the tumour and minimal side effects on surrounding organs is highly complex. To address this, the IMRT planning process is typically split into three sequential sub-problems: beam angle optimisation (BAO), fluence map optimisation (FMO), and multi-leaf collimator sequencing [5]. First, the BAO problem aims to identify the best possible combination of beam angles from which the radiation should be delivered, also known as the beam angle configuration (BAC). Once a BAC has been selected, the optimal intensities for that BAC must be found (Fluence Map Optimisation problem, FMO). Finally, in the MLC sequencing problem, we compute a set of deliverable aperture shapes and their corresponding intensities.

This sequential approach ends with a treatment plan consisting of a large set of aperture shapes (with corresponding intensity values). Unfortunately, having too many apertures and larger intensity values per aperture means longer treatment time. The total delivery time of a treatment plan is calculated considering both the *beam-on* time and the decomposition times. The beam-on time (BoT) is the total time a patient is exposed to radiation. The decomposition time is the time the linear accelerator needs to move from one bean angle in a BAC to the next one and the time needed by the MLC to move from one aperture shape to the other [6,7].

As a general rule, prolonged treatment time is something we want to avoid, as it increases the attention time per patient and, thus, reduces the number of patients treated per day [8]. Further, longer treatment plans are more likely to suffer from inaccuracies produced, for instance, by patient’s movements.

One strategy commonly used to minimise the total delivery time of treatment plans generated using the sequential approach described before is to reduce the number of apertures. This can be made by “rounding” the intensity values computed during the FMO phase. Unfortunately, such strategies can severely impair the final treatment plan quality.

One alternative to the sequential approach that does not require any “rounding” process is the direct aperture optimisation problem (DAO). The main idea in DAO is to solve the FMO problem considering a limited number of deliverable aperture shapes and the physical constraints associated with the MLC sequencing.

To solve the DAO problem, we must find a set of aperture shapes and their associated intensity values [9]. Usually, aperture shapes are optimised using heuristic strategies [10,11] or looking for the best possible combination of aperture shapes from a pre-defined set of apertures [12]. To optimise intensity values, gradient-based optimisation methods are usually implemented. Compared to the sequential approach, the treatment plan obtained using DAO is not only deliverable, but also better regarding the objective function value [13].

In this paper, we implement a particle swarm optimisation algorithm (PSO) combined with a mathematical programming technique to solve the DAO problem. PSO is recognised for effectively solving large-scale nonlinear optimisation problems through a good balance between exploitation (local search) and exploration (global search) [14,15]. While the PSO algorithm finds the best aperture shapes at each beam angle for a given BAC, the mathematical programming algorithm optimises each aperture’s intensity value. Also, we present a reparation heuristic for those aperture shapes that have a negligible effect on the treatment plan. To analyse our algorithm results, we use a set of clinical cases of prostate cancer and compare the treatment plans obtained by our algorithm to those obtained by the traditional sequential approach. The results show that our algorithm can find deliverable treatment plans using fewer apertures and significantly reduce the beam-on time compared to the traditional sequential approach. Compared to deliverable treatment plans with a similar number of apertures, our algorithm outperforms them regarding objective function values.

The remainder of this paper is organised as follows: Section 2 introduces the general concepts of IMRT and DAO and the mathematical models we will consider in this study. In Section 3, the algorithms we implement in this paper are presented. Section 4 presents the results obtained by our algorithm applied to a prostate case. A discussion of these results is also included in this section. Finally, in Section 5, we draw the main conclusions of our work and outline future work.

## 2. IMRT and the DAO Problem

In this section, we first discuss the main features of the IMRT problem and how to model it. Then, we introduce the DAO problem and present a brief literature review, focusing on the algorithms that have been previously proposed to solve the DAO problem.

### 2.1. Intensity Modulated Radiation Therapy

**Figure 2:**
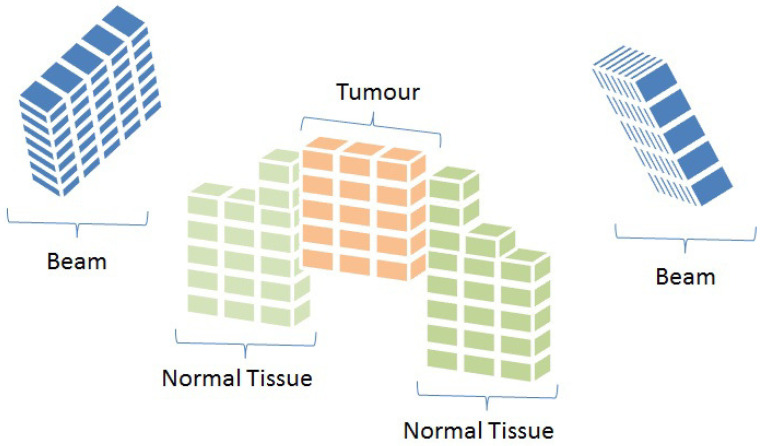
Representation of beam angles and organs discretised into beamlets and voxels, respectively (Cabrera-Guerrero et al. [17]).

To mathematically model the IMRT problem, we first need to discretise each beam angle into *beamlets*, and each region (tissues and tumour) into a set of small sub-volumes called *voxels* [16]. See Figure 2 for a graphical representation of these concepts.

**Figure 3:**
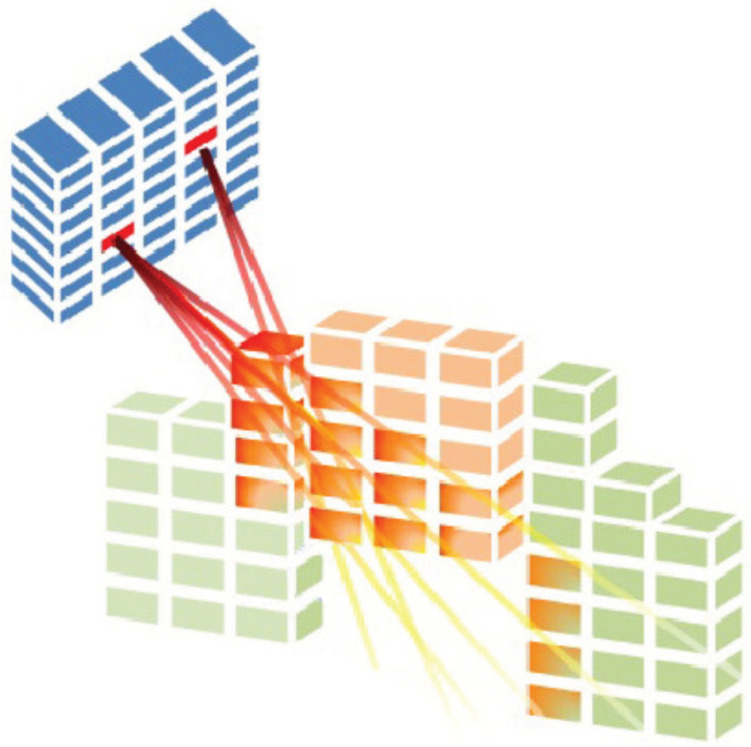
Radiation is delivered from a subset of beamlets, and it irradiates voxels at both tumour and organs at risk (Cabrera-Guerrero et al. [17]).

Thus, the IMRT problem can be modelled using the representation depicted in Figure 2 [12,17,18,19,20,21]. First, we model the dose distribution deposited in the voxels that compose a region. As mentioned above, beam angles are divided into a set of *n* beamlets, being *n*, the total number of beamlets summed over all the possible beam angles. Let A be a BAC and $x\in R_{≧0}^{n}$ be an intensity vector or fluence map solution for A. Each vector component $x_{b}$ represents the length of time the patient is exposed to the radiation of the *b*-th beamlet. The radiation dose deposited into each voxel *v* of region *r* by fluence map *x* is computed by the expression [16,20]

(1) $$d_{v}^{r}\left(x\right)=\sum_{b=1}^{n}(D_{v1i}^{r}x_{b})\;\forall v=1,2,\ldots ,m^{r},$$

where $m^{r}$ is the total number of voxels in the region *r*, $r\in R=\{O_{1},\ldots ,O_{Q},T\}$ is an element of the index set of regions, with the tumour indexed by r=T and the organs at risk and normal tissue indexed by $r=O_{q}$ with q=1,…,Q. $D^{r}\in R^{m^{r}\times n}$ is the dose deposition matrix related to region *r*, where $D_{vb}^{r}≧0$ defines the rate at which the radiation dose along beamlet *b* is deposited into voxel *v* of region *r* (As shown Figure 3). The set $X\left(A\right)\subseteq R^{n}$ is the set of all feasible fluence maps when the BAC A is considered. Note that searching for an optimal fluence map *x* over the X(A) space implies solving the FMO problem.

Based on the dose distribution in Equation (1), physical and biological models have been proposed in the literature (see Ehrgott et al. [16] for a survey). This study uses the convex nonlinear penalty function in [22,23]. In this model, each voxel is penalised according to the squared difference between the actual and the prescribed doses. This formulation yields a quadratic programming problem with only linear non-negativity constraints on the fluence values [22]. This model is as follows:

(2) $$\begin{array}{c} \begin{array}{cc} & \min_{x}z\left(x\right)=\sum_{r\in R}\left[\frac{1}{m^{r}}\sum_{i=1}^{m^{r}}\left[{\underline{\lambda }}_{r}{\left(Y_{r}-d_{v}^{r}\left(x\right)\right)}_{+}^{2}+{\bar{\lambda }}_{r}{\left(d_{v}^{r}\left(x\right)-Y_{r}\right)}_{+}^{2}\right]\right]\; \end{array} \end{array}$$

where parameter $m^{r}$ is, again, the number of voxels of the region *r* and $Y_{r}$ is the desired dose for the voxels of the region *r*. The function ${\left(\cdot \right)}_{+}$ is the maximum between 0 and (·), $d_{v}^{r}\left(x\right)$ gives the dose delivered by fluence map *x* to voxel *v* of the region *r* (see Equation (1)), and ${\underline{\lambda }}_{r}$ and ${\bar{\lambda }}_{r}$ are the penalty weights parameter of under-dose and overdose related to region *r*, respectively. Since the Equation (2) is convex, the optimal fluence maps can be obtained using mathematical programming techniques.

### 2.2. Direct Aperture Optimisation

The Direct Aperture Optimisation [10] merges the FMO and MLC problems, optimising the fluence map considering the constraints imposed by the MLC device. This means that the decision variables we focus on are not the beamlet intensities (as we did in the FMO problem), but the beamlet apertures and their corresponding aperture intensities. One consequence of this change is that the model becomes a mixed integer nonlinear problem as the beamlet apertures are binary variables (open/closed). Having binary variables makes the problem too hard to be solved by mathematical programming techniques, as we used to do with the FMO problem.

**Figure 4:**
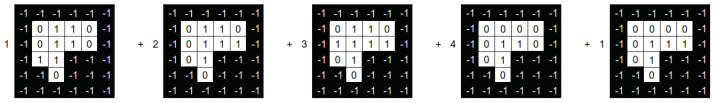
Set of aperture shapes and intensity values associated with a beam angle.

Let us consider a BAC $A=\{A_{1},\ldots ,A_{U}\}$, where $U\in N_{>0}$ represents the number of beams that are part of the BAC A. Consider that we represent a DAO solution as the set $H=\{\left(P^{1},I^{1}\right),\ldots ,\left(P^{N},I^{N}\right)\}$, where the $\left(P^{c},I^{c}\right)$ tuples correspond to a set of $\Theta^{c}$ aperture and intensity values for some beam angle *c*. We define each aperture shape $S_{i}^{c}\in P^{c}$ as a matrix of binary variables. Figure 4 gives an example of a tuple $\left(P^{c},I^{c}\right)$ for a beam angle *c*.

As we can see, the value of an element in the matrix is 1 if the radiation passes through the associated beamlet and 0 otherwise. The elements with value −1 are not considered, as the associated beamlets do not hit any voxel from the tumour. Also note that because of MLC physical constraints, the matrix $S_{i}^{c}$ is a consecutive 1’s matrix (C1), that is, for each row, 1 values must be consecutive, with no 0 value in between them.

To evaluate z(x), it is necessary to obtain the fluence map *x*, used in Equation (1), from the DAO solution. To this end, we first need to compute an aggregated matrix for each tuple in *H*. This aggregated matrix can be obtained through a positive linear combination of the aperture shapes $S_{i}^{c}$ and their corresponding intensities $I_{i}^{c}$ for angle A:

(3) $$A_{c}=\sum_{i=1}^{\Theta^{c}}S_{i}^{c}\cdot I_{i}^{c}\;$$

**Figure 5:**
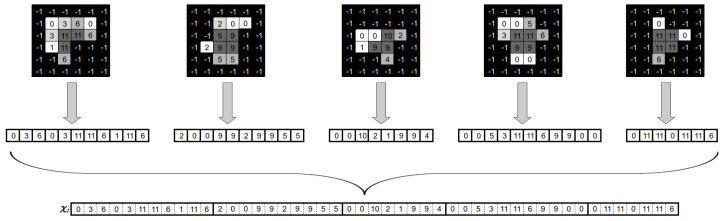
Generation of a fluence map from an angle’s apertures and associated intensities.

Then, we need to convert the aggregated matrix $A_{c}$ obtained in Equation (3) to a fluence map *x* vector. We perform this by mapping the position of each beamlet in the aggregated matrix of beam angle A to its corresponding position *b* in the fluence map solution *x* of beam angle A. Figure 5 shows how to do this.

#### Direct Aperture Optimisation Related Work

The DAO problem was first introduced by Shepard et al. [10]. In their paper, the authors identify as input of the problem the beam angles, the beam energies, and the number of apertures per beam angle. At the same time, the decision variables are the aperture shapes and their intensities. Currently, several different techniques have been used to solve the DAO problem. Some of these techniques are classified as stochastic search methods. These methods apply small changes in the leaf position of the apertures. When a change in the leaf position improves the objective function, it is accepted. It is important to remark that the changes in this method are stochastic [3,10,11,12,21,24,25,26,27].

Other methods for solving the DAO problem are based on gradient leaf refinement. In these methods, the leaf position is used as the optimisation variable. The relationship between the objective function and the leaf position is established, and the first derivative is given. Such algorithms have been applied to various commercial therapeutic systems, including the direct machine parameter optimisation model used in Pinnacle and RayStation systems [28,29]. Column generation methods have also been proposed in the literature [9,30,31,32,33]. In these methods, the initial apertures are not set at the beginning of an iteration; instead, deliverable apertures are individually added to the treatment plan. The iteration process involves two steps. First, the price problem is solved to generate the deliverable aperture that can improve the objective function, which is added to the treatment plan. Then, the new set of aperture weights is optimised in the master problem.

Unfortunately, the methods above also suffer from some issues. For instance, column generation approaches usually converge very fast; however, they do not allow for a hard limit on the number of apertures, which may translate to unreasonably long total treatment times and negligibly small apertures [34]. A relevant issue in stochastic search and gradient-based leaf refinement techniques is generating the initial solution. The quality of the initial solution influences the quality of the given final solution, as seen in [12,24].

All in all, solving the DAO problem using a limited number of apertures and obtaining good objective quality function values is an open problem that is worth to be studied.

## 3. Solution Method

This section introduces our hybrid PSO algorithm to solve the DAO problem. The main goal of our algorithm is to obtain a high-quality treatment plan for IMRT that consists of a set of deliverable set of aperture shapes and their corresponding intensity values.

In Section 3.1, we explain the original PSO algorithm proposed in [35] and how we adapt it to the DAO problem. Then, in Section 3.2, we define a reparation heuristic that uses a mathematical programming algorithm to improve the solution found by our PSO algorithm.

### 3.1. Particle Swarm Optimisation

The PSO is a nature-inspired population-based metaheuristic algorithm that imitates the social behaviour of birds in nature. This swarm consists of particles that search the objective space intending to find different high-quality solutions. Each particle is, in turn, composed of two fitness-related elements. The first element is the current fitness value of the *i*-th particle, and the second element is the fitness value of the best position the *i*-th particle has ever found during the algorithm execution, $pbest_{i}$. Finally, the algorithm also keeps track of the best fitness value found so far, gbest.

The PSO starts with an initial population of particles whose positions have been randomly assigned. The *i*-th particle’s position at iteration *t* is represented by $x_{i}^{t}$. The direction of particles in each iteration is determined by a velocity variable denoted by $v_{i}^{t}$ that obtains its value from Equation (4).

(4) $$v_{i}^{t+1}=cf*\left(wv_{i}^{t}+c_{1}r_{1}\left(pbest_{i}-x_{i}^{t}\right)+c_{2}r_{2}\left(gbest-x_{i}^{t}\right)\right),$$

where *t* is the current iteration, $pbest_{i}$ is the best position the *i*-th particle has achieved, and gbest is the best position any particle in the swarm has achieved. Parameter cf is the constriction factor used to adjust the velocity of each particle and obtain a balance between exploration and exploitation. The parameter *w* is the algorithm’s inertia and controls the last velocity contribution. Parameter $c_{1}$ and $c_{2}$ are learning factors for managing the impact of $pbest_{i}$ and gbest. Parameters $r_{1}$ and $r_{2}$ are random numbers between 0 and 1. The new position of each particle is updated by adding the current velocity to the function of the position of the particle, as shown in Equation (5).

(5) $$x_{i}^{t+1}=x_{i}^{t}+v_{i}^{t+1}$$

**Figure 6:**
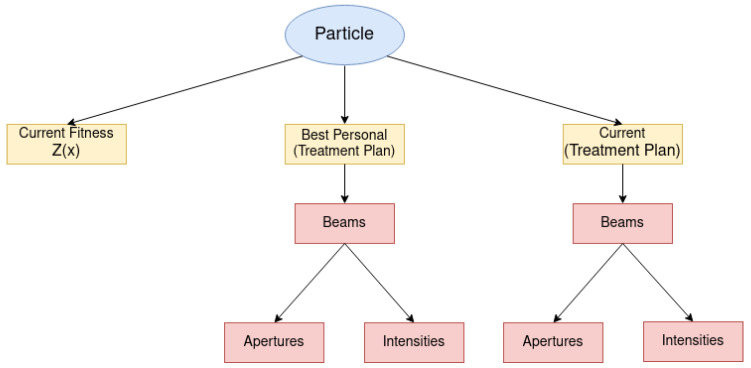
DAO solution on particle representation.

In the proposed algorithm, we represented the particles as shown in Figure 6. As we can see, the particle is composed of three attributes, namely the current fitness value (a real-valued attribute), its best singular position (a treatment plan), and its current position (a treatment plan). Naturally, the current fitness value results from evaluating the current particle’s position in the objective function considered by the algorithm.

As in any other heuristic algorithm, solutions generated by the PSO algorithms are not (necessarily) optimal. One drawback of the PSO implemented here is that there is no relation between the intensity values associated with an aperture and the aperture itself. Unfortunately, as mentioned before, the aperture shape optimisation problem is an NP-hard problem that mathematical programming solvers cannot solve in a reasonable time. Unlike this, the apertures’ intensity optimisation problem (also known as aperture weight optimisation [9] or segmentation weight optimisation) is a convex continuous problem that can quickly be solved for solvers such as Gurobi (see, for instance, [24,25,26]). Then, we propose to implement a hybrid PSO with a mathematical programming algorithm to solve the DAO problem. We use the PSO algorithm to find a set of aperture shapes and their corresponding intensities, which the linear solver will then optimise.

**Figure 7:**
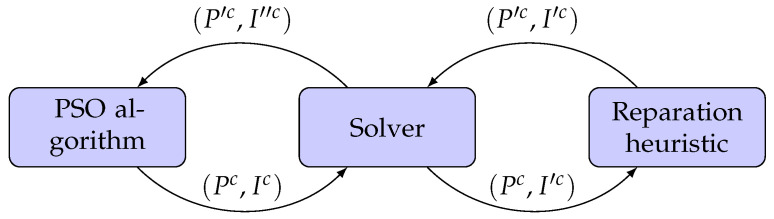
Interaction between PSO algorithm, linear solver and reparation heuristic.

To better understand the algorithm’s behaviour, we can see in Figure 7 how an aperture shape and the associated intensities change in each step. Considering the representation of the treatment plan mentioned in Section 2.2, we can represent the aperture shape and the intensities obtained by the PSO algorithm like a tuple $\left(P^{c},\;I^{c}\right)$. The intensities $I^{c}$ are optimised by the solver at the end of each iteration of the PSO algorithm. As a result, we obtain a new tuple $\left(P^{c},\;I^{\prime c}\right)$ where, as mentioned before, some intensities in $I^{\prime c}$ are set to zero by the solver. To improve the aperture shapes that resulted in (near) zero intensity value after the solver optimisation, we use a reparation heuristic. This heuristic only modifies $P^{c}$, leading to a new tuple $\left(P^{\prime c},\;I^{\prime c}\right)$. Finally, the reparation heuristic passes on the Solver the tuple $\left(P^{\prime c},\;I^{\prime c}\right)$ so we can obtain the optimal intensity values for the new set of apertures $P^{\prime c}$, generating the tuple $\left(P^{\prime c},\;I^{\prime \prime c}\right)$. Finally, the treatment plan defined by the tuple $\left(P^{\prime c},\;I^{\prime \prime c}\right)$ is passed onto the PSO algorithm for the next iteration. This process is repeated until the PSO algorithm meets some termination criterion (e.g., it reaches a predetermined number of iterations).

### 3.2. Reparation Heuristic

As mentioned in the previous paragraph, as a result of the solver usage, we obtain the optimal intensities for each aperture at each beam angle. Since the optimisation solver is conditioned to the aperture shapes obtained at each iteration by the PSO algorithm, it is not unusual that some of the intensities end up in the optimisation process with values close to zero.

In practice, apertures with associated intensities near to zero value are equivalent to having no aperture at all, i.e., an insignificant (or null) impact on the treatment plan. Further, improving the shapes of those apertures with intensity values close to zero is complex. To address this issue, we propose a reparation heuristic that allows us to avoid (as much as possible) those apertures with a negligible effect on the treatment plan.

**Figure 8:**
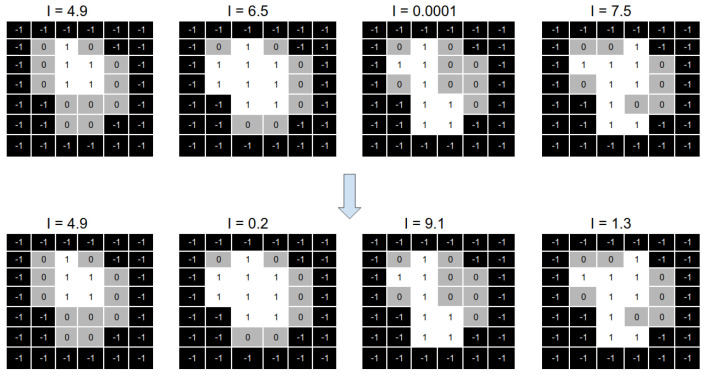
Representation of the change in the intensities of a set of apertures using the solver.

Figure 8 shows a numerical example of the intensities optimisation process. On top of the image, we can see four aperture shapes with their associated intensities. We can see that all the intensities are modified on the bottom part of the same image.

The main idea of the reparation heuristic proposed here is to replace those apertures with intensity values closer to zero with apertures that (hopefully) can help after running the solver. Particularly, we aim to irradiate those parts of the aperture shape that are not irradiated from any other aperture of the beam angle.

**Figure 9:**
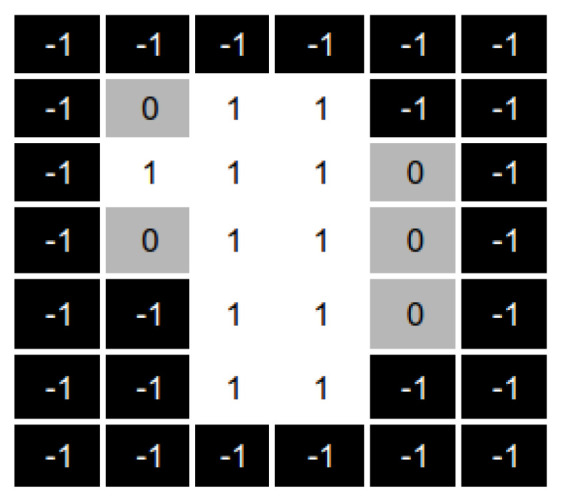
Overlapping matrix from the apertures with intensities over one.

To this end, we generate a new aperture that results from overlapping the apertures with an intensity value greater than 1. We call this new aperture the “overlapped aperture”, and the ones with intensity values greater than one “the original apertures”. Figure 9 shows an example of the apertures overlapping process. Fields with a value of 1 correspond to beamlets radiation passes through. Fields with zero value correspond to the beamlets closed in the original apertures. Finally, −1 corresponds to the inactive beamlets (those that do not hit the tumour).

As shown in Figure 9, the overlapped aperture corresponds to the original apertures’ aggregation, i.e., the overlapped aperture keeps open beamlets that are open in at least one original aperture and sets closed those beamlets that are closed in all the original apertures.

As a result of this aggregation process, we have a matrix showing all the beamlets currently open in at least one original aperture. As mentioned above, we want to diversify our search, and thus, we want to irradiate from those fields that are not currently in use.

**Figure 10:**
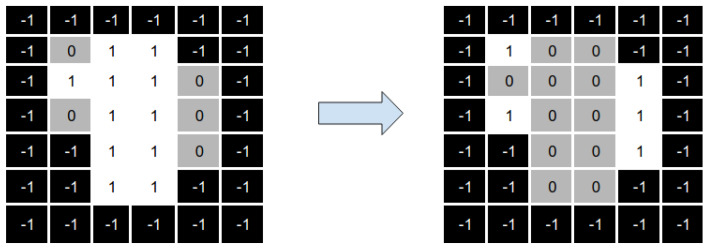
Complementary matrix generated from the overlapping matrix.

To this end, the reparation heuristic generates the complementary matrix of the overlapped matrix, as shown in Figure 10.

**Figure 11:**
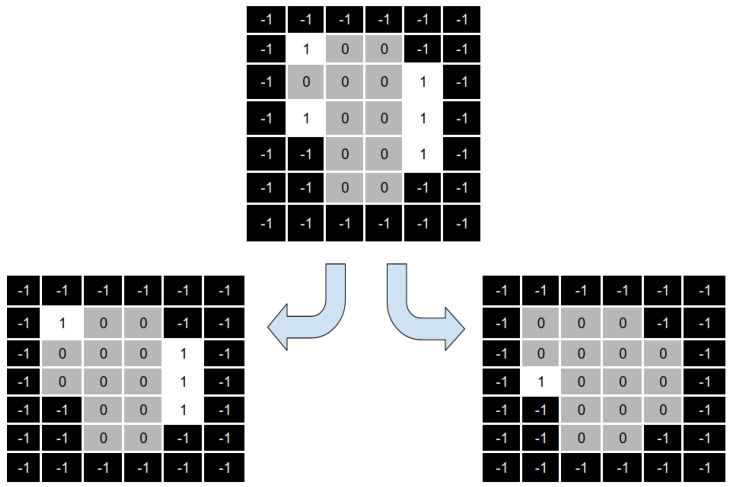
Dividing the complementary matrix so we can obtain deliverable aperture shapes.

It is important to keep in mind some considerations about the application of our reparation heuristic. First, in some cases, the shape of the complementary matrix does not satisfy the MLC physical constraints and can not directly replace the original aperture. In that case, we can select part of the aperture that is actually deliverable and remove those parts that do not satisfy MLC physical constraints. As shown in Figure 11, we divide the complementary matrix into two different apertures that satisfy the MLC physical constraints.

Second, suppose the number of original apertures with an intensity value close to zero is more than one. In that case, we must divide the complimentary matrix to generate as many new apertures as needed. Note that this situation can help us to solve our first consideration (undeliverable aperture shapes), as we can divide the complementary matrix in such a way that all (or most of) the open beamlets in the complementary matrix can be added to the new apertures (see, for instance, Figure 11).

**Figure 12:**
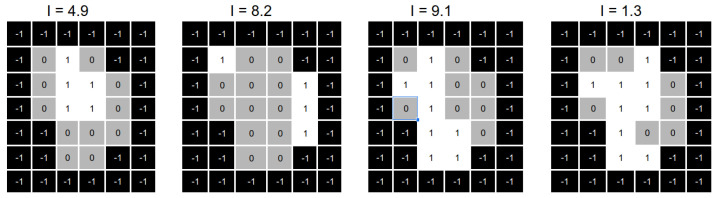
Representation of the apertures obtained after the reparation process.

Finally, the reparation heuristic replaces those apertures with (near) zero intensity values by the aperture shapes obtained in the previous step. We need to note that, in some cases, one or more apertures still with (near) zero intensity values as the number of deliverable aperture shapes produced by the reparation heuristic is less than the number of apertures with (near) zero intensity values. Once we obtained the repaired aperture shapes, we optimised the intensities values, as shown in Figure 12.

## 4. Computational Experiments

This Section introduces the experiments performed by our algorithm and analyses the obtained results. The Section is divided into three subsections. In Section 4.1, we introduce the set of instances considered in our study and the parameters used by the PSO. In Section 4.2, we obtain the best parameters for the PSO algorithm using the framework Irace [36]. Finally, in Section 4.3, we compare our PSO to two algorithms used in the literature. Comparison is made regarding the obtained objective function values, the required number of aperture shapes, and their beam-on time.

### 4.1. Experimental Setup

**Figure 13:**
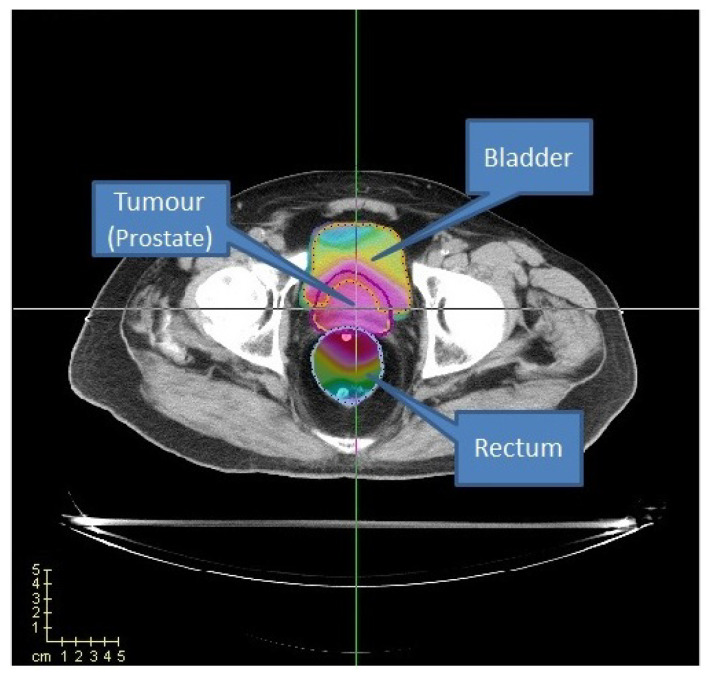
Prostate case from CERR. Two OARs (bladder and rectum) are considered.

In this work, we perform a set of initial experiments on the prostate case instance from *CERR package* [37] and also examine a prostate case acquired from Clinica Alemana de Santiago, Chile. This particular patient is denoted as TRT001 [19]. We use this prostate case to evaluate the performance of the PSO algorithm introduced in Section 3.1. For the CERR and TRT001 cases, we consider three organs: the prostate, where the tumour is located, the bladder, and the rectum (see Figure 13). We label the rectum and the bladder as organs at risk (OARs) and the prostate as planning target volume (PTV).

The number of voxels per region in the CERR case is 15,172 for the prostate, 22,936 for the bladder and 18,128 for the rectum. We consider 72 beam angles, all of which are on the same plane. Similarly, in the TRT001 case, the prostate comprises 13,081 voxels, the bladder holds 19,762 voxels, and the rectum encompasses 8500 voxels.

Like other works in the problem we consider a set of 14 equidistant BACs [12,17,18,21,24,25]. Each BAC consists of five beam angles for the CERR and TRT01 instances, as shown in Table 1.

Table 2 details the prescribed doses, $Y_{r}$, considered per each organ at all the instances and the weights for both under-dose ${\underline{\lambda }}_{r}$ and overdose ${\bar{\lambda }}_{r}$.

### 4.2. Irace Parameter

To optimise the parameter used in the PSO algorithm implemented, we tried the package Irace [36]. This package is an extension of the iterative F-race algorithm (I/F race) [38,39]. The principal use of this method is for the automatic configuration of optimisation algorithms. This is performed by finding the most appropriate configuration of parameters from a set of instances executed in the algorithm. This package has also been used for the parameters optimisation of the algorithm proposed by Caceres et al. [21]. That said, using IRace aims to find suitable parameters for our PSO implementation. The parameters to optimise within the IRace package are shown in Table 3.

Table 4 shows the results provided by the IRace package:

The number of iterations used by our algorithm is given by Equation (6), where we set the evaluation to 40,000 (number obtained testing the algorithm) and an Npop of 518 (given in Table 4) doing a total of 95 iterations, like limits for the algorithm.

(6) $$Iterations=\frac{evaluation}{Npop}.$$

### 4.3. Experiments on Test Instances

In our experiments, we measure the performance of the proposed PSO using the best-found parameter configuration, described in Section 4.2. Note that we run our algorithm 30 times per BAC, as 30 is a widely accepted value for statistical analysis [40].

Table 5, Table 6, Table 7 and Table 8 report the results obtained by both the sequential and the PSO approaches when applied to the CERR and TRT001 cases. As mentioned in the previous section, the IMRT sequential approach obtains a fluence map, optimising the dose-volume model of the FMO problem. Next, the MLC sequencing problem is solved for the resulting fluence maps by using a well-known algorithm from [7], which finds a set of apertures that minimise the BoT. In Table 5 and Table 6, column z(x∗) corresponds to the cost of the optimal fluence map using the function in Equation (2). Columns z(r(x∗)), $z(r_{2}\left(x*\right))$ and $z(r_{4}\left(x*\right))$ correspond to the cost of the fluence maps with intensities rounded to the nearest integer, the nearest multiple of 2, and the nearest multiple of 4, respectively. For each rounding, we also report the number of apertures generated by the MLC sequencing algorithm (#ap) and the BoT.

Table 7 and Table 8 report the results obtained by our PSO algorithm. Due to its stochastic nature, the strategy was run 30 times on each instance. We report the mean over the 14 instances of each set, the best value for each set, the mean number of apertures with intensity different to zero, and the mean BoT. We need to point out that apertures for which the intensity is set to zero by the mathematical programming solver in the last iteration are considered closed.

When comparing the objective function value reported by the PSO and the optimal (but not deliverable) fluence map, the difference is 29.41% and 30.49% for CERR and TRT001, respectively, with the PSO algorithm being the one with the higher objective value. This difference in the objective function value is reduced when the rounding process is applied to the optimal fluence map. For instance, when the optimal fluence map is rounded to the nearest multiple of 1$\left(z\left(r\left(x^{*}\right)\right)\right)$ and 2$\left(z\left(r_{2}\left(x^{*}\right)\right)\right)$, the difference is 25.39% and 13.71% for the CERR case and 26.98% and 17.98% for the TRT001 case, respectively. Further, rounding to the nearest multiple of 4$\left(z\left(r_{4}\left(x^{*}\right)\right)\right)$ leads to an impairment in the quality of the rounded treatment plan that makes solutions provided by our PSO algorithm become better in all cases. Further, even though our algorithm is not better than the $r_{1}\left(x^{*}\right)$ and $r_{2}\left(x^{*}\right)$ treatment plans (with respect to the objective function value), the number of aperture shapes our solutions need is always smaller than the apertures needed by the solutions obtained by the sequential approach. Also, it is interesting to note that even though our approach is not directly focused on reducing the beam on time value, our approach reports better values in all cases compared to the sequential approach. This is mainly because of the fact that we use far fewer aperture shapes in our final treatment plans.

**Figure 14:**
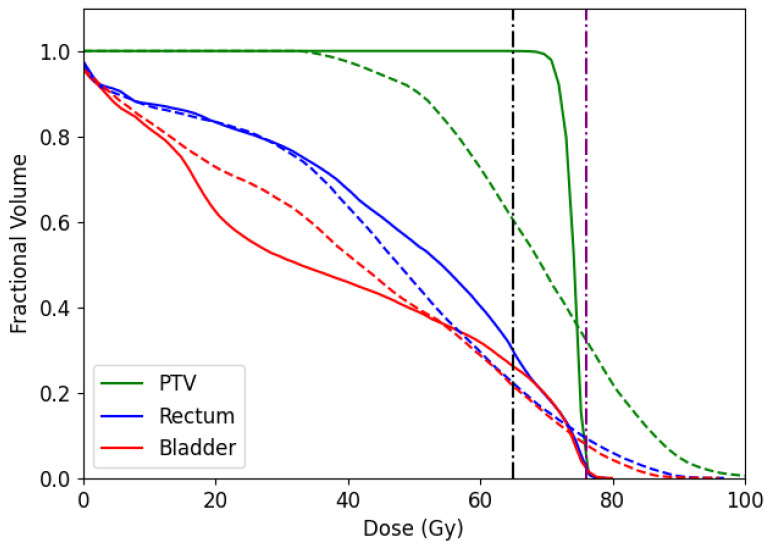
Dose-volume histogram comparing dose obtained by PSO algorithm (solid line) and optimal fluence map obtained by FMO (dashed line) for a prescribed dose of 76 Gy to PTV, and 65 Gy to the rectum and bladder (purple and black horizontal dashed-point line, respectively) with BAC 1 in CERR instance.

**Figure 15:**
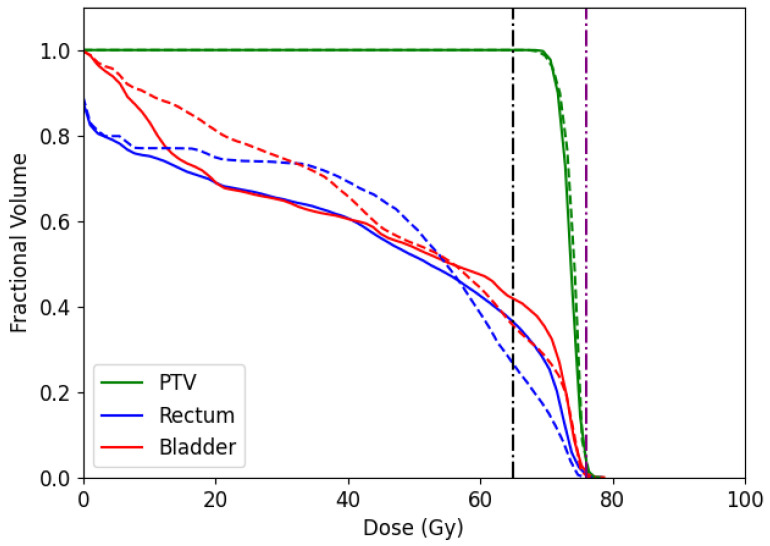
Dose-volume histogram comparing dose obtained by PSO algorithm (solid line) and optimal fluence map obtained by FMO (dashed line) for a prescribed dose of 76 Gy to PTV, and 65 Gy to the rectum and bladder (purple and black horizontal dashed-point line, respectively) with BAC 1 in TRT001 instance.

In addition, we report the dose-volume histogram (DVH) for the CERR and TRT001 in Figure 14 and Figure 15, respectively. DVH curves specify the received dose level by different volumes of structures. In the case of CERR, we can see that our algorithm obtains treatments that do not overdose the voxels in the PTV. Unlike this, the solutions obtained by the optimal fluence map overdose above 30% of PTV voxels. When observing the OARs, our algorithm overdoses more voxels than the optimal fluence map. However, the max overdose received for the voxels is less than the received by the optimal fluence map. In the case of TRT001, the PSO and the optimal fluence map have a similar curve, where both do not overdose the PTV. When observing the OARs, our algorithm overdoses more voxels than the optimal fluence map. It is necessary to remember that the optima fluence map is not a deliverable treatment and needs to pass for the MLC sequencing problem.

## 5. Conclusions

This paper introduces a hybrid heuristic based on PSO and mathematical programming to solve the DAO problem in radiation therapy for cancer treatment. The proposed PSO heuristic finds a set of deliverable aperture shapes and their corresponding intensities for each beam angle within a clinically acceptable time. Further, even though our heuristic algorithm was allowed to use only five aperture shapes per beam angle, they could find very competitive treatment plans.

Comparing our algorithm with the traditional sequential approach shows that the proposed algorithm can obtain competitive results regarding the objective function value. However, the difference with the optimal solution generated by the FMO is still significant. On the opposite, when evaluating the number of apertures generated by our algorithm, we can observe a substantial reduction compared to the traditional approach. This is very important as fewer aperture shapes mean, in general, shorter treatment times, which is something desirable from a clinical point of view.

In future work, we can see different research lines to improve the obtained results. First, we believe that improving the reparation heuristic to activate apertures that have intensities close to zero would allow us to find better-quality treatment plans. This is because the more apertures are used, the better the treatment plan quality. Note that, as mentioned before in the paper, this would be at the cost of longer treatment times. In addition, we seek to extend our single-objective PSO algorithm to a multi-objective one. This is because IMRT is an inherently multi-objective problem, since there is a compromise between tumour irradiation and avoiding damage to the organs at risk. Extending our approach to a multi-objective one is a challenging task from both computational and clinical points of view. However, we are sure that addressing the problem as a multi-objective one will help us better understand the underlying trade-offs between tumour control and OARs sparing.

## Figures and Tables

**Table 1:** Equidistant BACs and their corresponding number of beamlets for the CERR and TRT01 cases.

BAC	Beam Angles					# Beamleats	# Beamleats
	$\theta_{1}$	$\theta_{2}$	$\theta_{3}$	$\theta_{4}$	$\theta_{5}$	**CERR**	**TRT**
1	0	70	140	210	280	336	327
2	5	75	145	215	285	336	329
3	10	80	150	220	290	333	328
4	15	85	155	225	295	333	330
5	20	90	160	230	300	329	334
6	25	95	165	235	305	328	334
7	30	100	170	240	310	333	333
8	35	105	175	245	315	336	330
9	40	110	180	250	320	337	329
10	45	115	185	255	325	335	329
11	50	120	190	260	330	331	335
12	55	125	195	265	335	329	335
13	60	130	200	270	340	329	332
14	65	135	205	275	345	328	328

**Table 2:** Value of $T_{i}$, ${\underline{\lambda }}_{i}$ and ${\bar{\lambda }}_{i}$ for function z(x).

Organ	$Y_{r}$	${\underline{\lambda }}_{r}$	${\bar{\lambda }}_{r}$
PTV	76 Gy	5	5
Rectum	65 Gy	0	1
Bladder	65 Gy	0	1

**Table 3:** Parameters of PSO used in Irace.

Parameter	Description	Range
*Npop*	Number of population	*Npop* $\in \left[100,600\right]$
$c1_{a}$	Local Learning factor on Apertures	$c1_{a}\in \left[0,2\right]$
$c2_{a}$	Global Learning factor on Apertures	$c2_{a}\in \left[0,2\right]$
$w_{a}$	Inertia weight on Apertures	$w_{a}\in \left[0,2\right]$
$cf_{a}$	Constriction factor on Aperture	$cf_{a}\in \left[0,2\right]$
$c1_{i}$	Local Learning factor on Intensities	$c1_{i}\in \left[0,2\right]$
$c2_{i}$	Global Learning factor on Intensities	$c2_{i}\in \left[0,2\right]$
$w_{i}$	Inertia weight on Intensities	$w_{i}\in \left[0,2\right]$
$cf_{i}$	Constriction factor on Intensities	$cf_{i}\in \left[0,2\right]$

**Table 4:** Best parameters’ values obtained by IRace.

Parameter	Value
Npop	418
$c1_{a}$	1.8751
$c2_{a}$	0.2134
$w_{a}$	0.5774
$cf_{a}$	1.6641
$c1_{i}$	0.3158
$c2_{i}$	1.7017
$w_{i}$	0.5331
$cf_{i}$	1.2389

**Table 5:** Results reported by the traditional two-step approach in the CERR dataset.

BAC	z(x∗)	z(r(x∗))	# ap	BoT	$z(r_{2}\left(x*\right))$	# ap	BoT	$z(r_{4}\left(x*\right))$	# ap	BoT
1	42.98	44.84	140	196	49.29	87	192	61.54	51	204
2	43.40	43.40	140	215	48.76	84	212	61.72	52	224
3	43.70	44.98	144	203	48.83	87	202	72.87	49	208
4	43.53	45.06	145	206	51.77	89	208	66.48	50	212
5	43.23	44.55	142	200	47.40	89	202	67.48	51	204
6	43.05	44.47	149	212	49.23	90	208	66.05	50	208
7	42.86	44.48	152	212	48.05	96	214	62.96	49	212
8	43.06	44.70	146	197	48.00	88	196	61.75	48	196
9	43.66	45.03	141	186	50.62	83	190	70.76	46	192
10	44.14	45.71	144	200	51.21	89	204	59.64	47	200
11	43.83	45.02	138	190	51.97	86	190	68.84	47	200
12	43.31	44.35	144	214	47.38	94	212	64.03	55	228
13	42.84	44.98	157	229	49.05	98	226	82.49	56	232
14	42.85	44.24	142	217	48.57	92	214	68.45	51	220
Average	43.32	44.71	144	205	49.30	89	205	66.80	50	210

**Table 6:** Results reported by the traditional two-step approach in patient TRT001 in the CAS dataset.

BAC	z(x∗)	z(r(x∗))	# ap	BoT	$z(r_{2}\left(x*\right))$	# ap	BoT	$z(r_{4}\left(x*\right))$	# ap	BoT
1	55.78	56.92	146	220	63.05	92	222	89.97	52	224
2	56.35	58.38	138	212	63.66	89	212	84.22	49	212
3	56.72	58.39	141	211	63.55	85	210	77.01	49	216
4	56.55	57.99	132	210	63.22	88	220	74.48	51	224
5	55.98	57.97	138	210	64.31	90	214	81.44	49	220
6	55.19	56.37	139	208	59.81	87	204	80.24	47	196
7	55.21	56.54	129	192	59.55	78	192	78.24	44	196
8	56.14	57.26	131	187	62.71	84	188	82.21	46	188
9	56.62	58.13	136	218	62.58	88	210	76.24	52	216
10	56.94	58.30	140	207	63.59	85	206	92.40	50	212
11	56.74	58.27	152	231	61.47	100	234	84.80	56	232
12	56.17	57.99	144	218	61.18	96	218	79.20	54	216
13	55.32	57.54	134	204	59.75	87	204	76.08	49	204
14	55.46	56.85	142	212	59.99	95	214	82.88	41	212
Average	56.08	57.64	138	210	62.03	88	210	81.39	49	212

**Table 7:** Results reported by the PSO algorithm in the CERR dataset.

BAC	z(x∗)	# ap	BoT
1	56.34	11.70	63.47
2	57.49	13.07	64.13
3	57.39	12.63	61.31
4	57.33	12.67	61.60
5	56.24	12.13	65.38
6	54.76	12.59	60.72
7	54.38	12.60	62.46
8	54.57	12.57	66.49
9	57.53	12.29	61.28
10	57.36	11.67	64.57
11	56.18	12.75	68.62
12	54.96	12.35	61.54
13	55.85	12.25	60.35
14	54.41	12.60	62.49
Average	56.06	12.42	63.17

**Table 8:** Results reported by the PSO algorithm in the TRT001 dataset.

BAC	z(x∗)	# ap	BoT
1	71.33	12.40	62.44
2	72.39	12.70	62.13
3	73.79	12.00	62.96
4	73.85	12.20	63.07
5	73.80	11.10	58.99
6	73.75	11.40	63.91
7	72.52	10.90	58.94
8	72.65	10.20	60.30
9	74.27	12.10	66.04
10	77.58	11.40	63.30
11	74.63	12.60	62.51
12	72.00	12.10	60.13
13	69.75	11.30	60.32
14	71.31	10.50	59.74
Average	73.18	11.64	61.77

## Data Availability

The data can be shared upon request.

## Abbreviations

The following abbreviations are used in this manuscript:

ASO	Aperture Segmentation Optimisation
AWO	Aperture Weight Optimisation
BAC	Beam angle configuration
BAO	Beam angle optimisation
BoT	Beam-on-Time
CERR	Computational Environment for Radiological Research
DAO	Direct Aperture Optimisation
DVH	Dose-Volume histogram
FMO	Fluence map optimisation
IMRT	Intensity modulated radiotherapy treatment
MLC	Multi-leaf Collimator
*Npop*	Population of solution
OAR	Organ at risk
PTV	Planning target volume
PSO	Particle Swarm Optimisation
SBRT	Stereotactic Body Radiation Therapy
VMAT	Volumetric Modulated Arc Therapy
